# Increased Calcium-Sensing Receptor Immunoreactivity in the Hippocampus of a Triple Transgenic Mouse Model of Alzheimer's Disease

**DOI:** 10.3389/fnins.2017.00081

**Published:** 2017-02-16

**Authors:** Emanuela Gardenal, Anna Chiarini, Ubaldo Armato, Ilaria Dal Prà, Alexei Verkhratsky, José J. Rodríguez

**Affiliations:** ^1^Human Histology and Embryology Unit, Medical School, University of VeronaVerona, Italy; ^2^Basque Foundation for Science, Achúcarro Basque Center for Neuroscience, IKERBASQUEBilbao, Spain; ^3^Department of Neuroscience, University of the Basque Country (UPV/EHU)Leioa, Spain; ^4^Faculty of Biology, Medicine and Health, The University of ManchesterManchester, UK

**Keywords:** Alzheimer's disease, β-amyloid, tau, calcium sensing receptor (CaSR), hippocampus

## Abstract

The Calcium-Sensing Receptor (CaSR) is a G-protein coupled, 7-transmembrane domain receptor ubiquitously expressed throughout the body, brain including. The role of CaSR in the CNS is not well understood; its expression is increasing during development, which has been implicated in memory formation and consolidation, and CaSR localization in nerve terminals has been related to synaptic plasticity and neurotransmission. There is an emerging evidence of CaSR involvement in neurodegenerative disorders and Alzheimer's disease (AD) in particular, where the over-production of β-amyloid peptides was reported to activate CaSR. In the present study, we performed CaSR immunohistochemical and densitometry analysis in the triple transgenic mouse model of AD (3xTg-AD). We found an increase in the expression of CaSR in hippocampal CA1 area and in dentate gyrus in the 3xTg-AD mice when compared to non-transgenic control animals. This increase was significant at 9 months of age and further increased at 12 and 18 months of age. This increase paralleled the accumulation of β-amyloid plaques with age. Increased expression of CaSR favors β-amyloidogenic pathway following direct interactions between β-amyloid and CaSR and hence may contribute to the pathological evolution of the AD. In the framework of this paradigm CaSR may represent a novel therapeutic target.

## Introduction

The calcium sensing receptor (CaSR) belongs to the extended family of plasmalemmal G protein-coupled heptahelical receptors (GPCRs); it shares the C subfamily of GPCRs with metabotropic glutamate receptors (mGluR) and γ-aminobutyric acid GABA_B_ receptors (Brown and MacLeod, 2001). CaSRs are widely distributed throughout the brain, with highest expression in the subfornical organ, hippocampus, striatum, cingulate cortex, cerebellum, ependymal zones of the cerebral ventricles and perivascular nerves around cerebral arteries, some CaSR expressing cells were found also in rat dorsal root ganglia (Ruat et al., 1995; Yano et al., 2004). The CaSR has been found abundantly expressed *in vivo* in neurons and in oligodendrocytes; *in vitro* studies demonstrated its expression in human primary astrocytes and in rat microglia (Ruat et al., 1995; Chattopadhyay et al., 1998, 1999a; Ferry et al., 2000; Dal Prà et al., 2005).

Numerous functions have been assigned to CaSR in the CNS from regulation of neuronal growth and migration, to the role in neurotransmission and synaptic plasticity (Ruat and Traiffort, 2013); CaSR can also contribute to astroglial functions, microglial reactivity and oligodendroglial development (Ruat and Traiffort, 2013). The level of extracellular ionized Ca^2+^ ([Ca^2+^]_o_) is usually considered to be stable in the brain, which is not really the case, since [Ca^2+^]_o_ undergoes rapid fluctuations in normal physiological processes such as development, synaptic transmission and aging as well as in pathological processes including neurodegeneration and Alzheimer's disease (AD; Small, 2009; Ruat and Traiffort, 2013).

Expression of CaSR markedly increases during development, specifically in perinatal and early post-natal periods just before and after birth (Chattopadhyay et al., 1997; Vizard et al., 2008). In the hippocampus CaSR was reported to regulate neuronal growth, as well as extension and branching of neurites (Vizard et al., 2008). In addition, CaSR has been identified in neocortical nerve terminals where it senses the [Ca^2+^]_o_ and activates voltage dependent non-selective cation channels (NSCCs) (Chen et al., 2010). It has been proposed that the decrease in [Ca^2+^]_o_ in the synaptic cleft may act as feedback to presynaptic CaSR and the associated increased activity of NSCCs may prolong action potentias; in this way CaSR may influence synaptic transmission through a homeostatic pathway to prevent synaptic failure when [Ca^2+^]_o_ falls (Ye et al., 1996). Studies *in vitro* conducted in the human astrocytoma cell line U87, and in primary cultures of rat microglia and oligodendroglia showed that CaSR stimulates Ca^2+^-activated K^+^ channels, thus contributing to local ionic homeostasis following the lowering of [Ca^2+^]_o_ due to increased neuronal activity; there are also indications that CaSR could also play a role in microglia activation (Chattopadhyay et al., 1998, 1999a,b). CaSR can form heterodimers with others GPCRs, like GABA_B_ receptors and mGluRs, which might be important for their trafficking to the membrane, their ligand binding sensitivity and thus the regulation of signaling responses (Gama et al., 2001; Chang et al., 2007). Activation of CaSR was also demonstrated to induce a distinct form of glutamate release independent on Ca^2+^ influx (Vyleta and Smith, 2011).

In the context of AD, CaSR has been reported to be directly activated by β-amyloid as well as by apoE (isoforms 3 and 4) (Conley et al., 2009). In rat hippocampal neurons exposure to β-amyloid stimulated openings of NSCCs linked to CaSR activation (Ye et al., 1997). How β-amyloid interacts with CaSR remains unclear, although it is possible that either β-amyloid fibrils or variously sized oligomers, having regular positive charges, mimic CaSR agonists, or hydrophobic interactions between β-amyloid and CaSR may take place (Ye et al., 1997). Thus, extracellular accumulation of β-amyloid can activate CaSR which, in turn, may contribute to Ca^2+^ dyshomeostasis observed in AD (Lim et al., 2014, 2015).

Previous studies on human cultured primary adult astrocytes showed that their exposure to exogenous β-amyloid_25–35_, a surrogate of β-amyloid_42_, triggers CaSR-dependent signaling cascade that stimulates the expression of nitric oxide synthase-2 (NOS-2) followed by an excessive release of nitric oxide (Chiarini et al., 2005), and increased expression of vascular endothelial growth factor (VEGF)-A (Chiarini et al., 2010). Most importantly, cultured human cortical astrocytes and neurons exposed to β-amyloid_25–35_, which binds and activates CaSR (Dal Prà et al., 2014), begin to excessively produce and secrete β-amyloid_42_ oligomers, contributing in this way to the β-amyloid load (Dal Prà et al., 2011; Armato et al., 2013).

In the present study, for the first time we aimed to determine *in vivo* CaSR expression in the 3xTg-AD mouse model and correlate its changes with β-amyloid load.

## Materials and methods

All animal procedures were carried out in accordance with the United Kingdom Animals (Scientific Procedures) Act of 1986 under the license from the Home Office. All efforts were made to reduce the number of animals by following the 3R's (reduction, refinement and replacement).

### Mice

Experiments were performed on male 3xTg-AD mice generated on 129/C57BL6 background (the wild type of which was employed as a non-Tg control). The 3xTG-AD mice harbor the mutant genes for amyloid precursor protein (APPSwe), presenilin 1 PS1M146V and tauP301L (Oddo et al., 2003a,b; Rodríguez et al., 2008). All 3xTg-AD and non-Tg control mice were obtained by crossing homozygous breeders. Animals were housed in groups of the same genotype and in the same cage according to their age, kept in 12 h light-dark cycles with *ad libitum* access to food and water.

The experimental groups chosen were of 9, 12, and 18 months of age, since it is at these ages that the amyloid and tau pathologies emerge resembling the human Alzheimer's disease progression. Indeed, the 3xTg-AD mice exhibit the highest intracellular β-amyloid deposition at 9 months of age, they start showing extracellular β-amyloid plaques followed by intracellular tau deposition at 12 months of age and they present massive Aβ plaques and neurofibrillary tangles at 18 months of age (Rodríguez et al., 2008; Olabarria et al., 2010).

### Fixation and tissue processing

As described previously (Olabarria et al., 2010; Kulijewicz-Nawrot et al., 2012) 3xTg-AD and non-Tg control mice at 9, 12, 18 months of age (Ns for non-Tg = 5, 5, 4; ns for 3xTg-AD = 4, 5, 3, respectively) were intraperitoneally anesthetized with sodium pentobarbital (50 mg/kg). Mice were trans-cardially perfused with 4% paraformaldehyde (PFA, Sigma, UK) and 0.1 M phosphate buffer (PB) at pH 7.4. Brains were removed and post-fixed in 4% paraformaldehyde overnight, then cut into 4–5 mm coronal slabs of tissue containing the entire rostrocaudal extent of the hippocampus, as previously described (Olabarria et al., 2011). The tissue was further sectioned in 40–50 μm-thick slices with a vibrating microtome (VT1000S, Leica, Milton Keynes, UK). Free floating sections were collected in 0.1 M PB and stored in a cryoprotectant solution containing 25% sucrose and 3.5% glycerol in 0.05 M PB at pH 7.4. Three coronal sections at levels −1.58 mm/−2.46 mm (dorsal hippocampus) posterior to Bregma, were selected from each animal for immunohistochemistry according to the mouse brain atlas of Paxinos and Franklin (2012).

### Antibodies

A mouse monoclonal anti-Calcium Sensing Receptor antibody (anti-CaSR; Sigma-Aldrich, UK; C0493) was used for the determination of CaSR expression throughout the dorsal hippocampus. For the identification of intracellular β-amyloid deposits and plaques a monoclonal mouse antibody that reacts with abnormally processed isoforms, as well as precursor forms of Aβ, recognizing an epitope within amino acids 3–8 (EFRHDS; anti-Aβ 6E10 [SIG-39320] Signet Laboratories, Dedham, MA) was used, for hyperphosphorylated Tau we employed the mouse monoclonal antibody anti-PHF Tau AT8 (Innogenetics, Gent, Belgium). For double immunostaining of CaSR with GFAP and Aβ, a rabbit anti-GFAP (Sigma-Aldrich, UK; G9269) and a mouse anti-Aβ Alexa 488 6E10 conjugated antibody (Covance, USA) were used. To assess for non-specific background labeling or cross reactivity between antibodies derived from different host species, a series of control experiments were performed, including the omission of primary (Supplementary Figure 1C) and/or secondary antibodies from the incubation solutions; resulting all of them in a total absence of target labeling.

### Immunohistochemistry

All the sections were processed at the same time using the same experimental conditions to minimize methodological variability (Noristani et al., 2010; Olabarria et al., 2011; Kulijewicz-Nawrot et al., 2012). Then sections were incubated for 30 min in 30% methanol in 0.1 M PB and 30% hydrogen peroxide (H_2_O_2_; Sigma, UK). Sections were then rinsed with 0.1 M PB for 5 min and placed in 1% sodium borohydride (Sigma, UK) for 30 min. The sections were then washed with PB profusely before rinsing in 0.1 M Trizma base saline (TS) for 10 min. Brain sections were then incubated in 0.5% bovine serum albumin (Sigma, UK) in 0.1 M TS and 0.25% Triton X-100 (Sigma, UK) for 30 min. Sections were incubated for 72 h at room temperature in primary antibody (mouse anti-CaSR, 1:250, Sigma, UK). The sections were rinsed in 0.1 M TS for 30 min and incubated in 1:400 dilutions of biotinylated horse anti-mouse IgG (Vector Laboratories, Peterborough, UK) for 1 h at room temperature. Sections were rinsed with 0.1 M TS for 30 min followed by incubation for 30 min in avidin-biotin peroxidase complex (Vector Laboratories, Peterborough, UK). The peroxidase reaction product was visualized by incubation in a solution containing 0.022% of 3,3' diaminobenzidine (DAB, Aldrich, Gilligham, UK) and 0.003% H_2_O_2_ for 7 min. The reaction was stopped by rinsing the sections in 0.1 M TS for 5 min followed by 0.1 M PB for 15 min. Brain sections were permanently mounted onto gelatinized slides and allowed to dry overnight. Sections were then dehydrated in ascending concentration of ethanol (50, 70, 80, 90, 95, and 100%) and, finally, xylene. Sections were then permanently mounted and coverslipped using Entellan (Merck KGaA, Darmstadt, Germany) and slides were left to dry overnight.

For the dual immunofluorescence labeling with CaSR and GFAP the sections were simultaneously incubated with both antibodies (mouse anti-CaSR, 1:250; rabbit anti-GFAP, 1:20,000) for 48 h at room temperature. Then CaSR and GFAP were detected in a sequential manner on the same sections by incubation of 2 h with Alexa Fluor 594-conjugated goat anti-mouse and Alexa Fluor 488-conjugated goat anti-rabbit (1:400, Invitrogen, Paisley, UK), respectively.

For the dual immunofluorescence labeling with CaSR and Aβ the sections were first incubated for 48 h at room temperature in CaSR antibody solution then in the Alexa 594 conjugated goat anti-mouse for 2 h, finally for 24 h in Alexa 488-conjugated mouse anti-Aβ antibody (1:1,000, Covance, USA).

Sections were rinsed with 0.1 M TS for 30 min and permanently mounted in an aqueous medium (Vectashield; Vector laboratories, Peterborough, UK).

### Optical density (OD) measurement

Using computer-assisted imaging analysis (ImageJ 2.0.0-rc-39/1.50b; NIH, USA) we analyzed the expression of CaSR by measuring its OD, as described previously (Cordero et al., 2005; Noristani et al., 2010, 2012). Briefly, to exclude any experimental errors and/or bias, all images were taken at constant light intensity with a Nikon Eclipse 80i microscope coupled with an 8001 MicroFIRE camera. Optical filters were used to ensure the specificity of the signal recorded by the camera. The OD was calculated from a relative scale of intensity ranking from 0 to 250, with a measurement of 250 corresponding to the area with very low CaSR expression and 0 corresponding to the area with highest labeling. The calibration density was kept constant for measuring all sections to avoid experimental variances. Non-specific OD in sections was measured from the corpus callosum (CC). CaSR density of the complete CA subfields of the hippocampus and their different layers (stratum pyramidale, PCL; stratum oriens, SO; stratum radiatum, S.Rad and stratum lacunosum-moleculare, S.Mol), except CA3 where we also studied stratum lucidum, were measured independently. Similarly, CaSR density of the dentate gyrus (DG) and its different layers (granular cell layer, GCL, molecular layer, ML and hilus) were measured individually. The OD of the region of interest is determined by outlining each layer and obtaining the mean value of the intensities in the selected area (Supplementary Figures 1A,B). The changes in CaSR density were analyzed against a constant control region (CC): 250 was divided by control region and the obtained factor was multiplied by the region of interest in every given section. Inverse OD (IOD) was obtained by subtracting the OD (after the control region normalization) from 250. Values of IOD were taken and averaged in both the left and right hemisphere of each slice. The results are shown as inverse optical CaSR density (IOD/pixel).

### Counting of Aβ-positive cells

β-amyloid containing neurons were counted in the entire extent of the CA1 region of the hippocampus, since this field shows the earliest and strongest accumulation of Aβ intracellular deposits. This quantification was carried out on six non-consecutive sections from dorsal hippocampus of three non-Tg and 3xTg-AD animals of 9 months of age immunolabeled with anti-Aβ 6E10 antibody.

### Statistical analysis

One-way analysis of variance (ANOVA) was used to determine changes in CaSR density between 3xTg-AD animals and their non-transgenic controls at different ages, followed by unpaired *t*-test comparisons at the different time points. Significance was accepted at *P* ≤ 0.05. The data were analyzed using GraphPad Prism software (La Jolla, CA, USA). Results are expressed as mean ± SEM.

## Results

**Figure 1:**
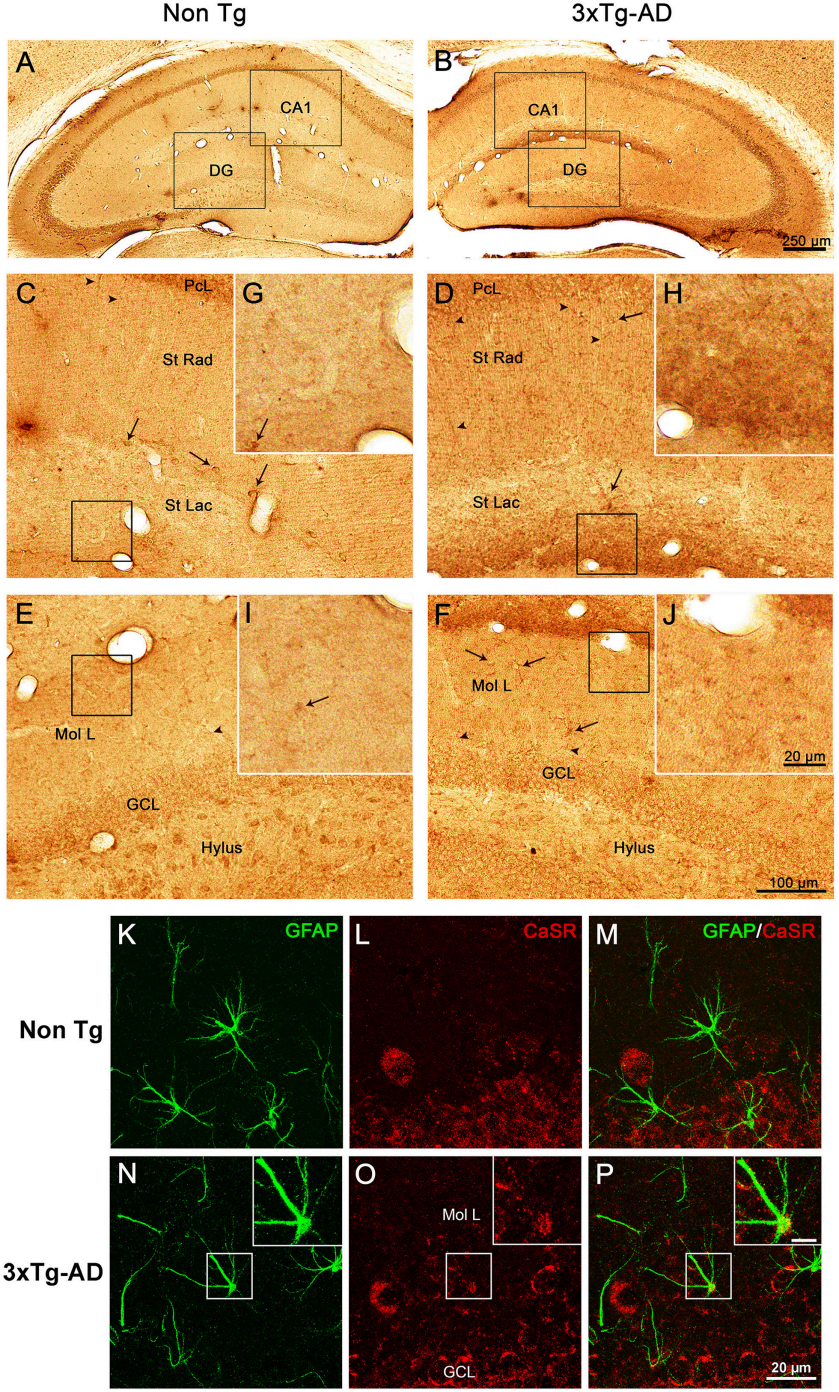
**CaSR expression and localization in hippocampus**. Brightfield micrographs showing the distribution of CaSR-IR within the dorsal hippocampus of 18 months old non-Tg controls **(A,C,E,G,I)** and 3xTg-AD mice (**B,D,F,H,J**; arrowheads show neuropil elements, whilst full arrows indicate interneurons). Confocal micrographs showing the immunoreactivity for CaSR (red) and for GFAP (green), in Non-Tg **(K–M)** and 3xTg-AD mice **(N–P)**. Inserts in **(N–P)** show an astrocyte bearing CaSR at higher magnification (scale bar 20 μm). CA1, Cornu Ammonis 1; DG, Dentate Gyrus; St Rad, Stratum Radiatum; St Lac, Stratum Lacunosum-Moleculare; Mol L, Molecular Layer; GCL, Granular Cell Layer.

Immunohistochemical labeling demonstrated that CaSR is present in the hippocampus of both 3xTg-AD and non-Tg control animals at all ages (9, 12, and 18 months) with a rather homogeneous distribution throughout all hippocampal areas (Figures 1A,B). Punctate CaSR labeling is mainly present in pyramidal neurons of the different CA subfields and in granule cells of the DG as well as in the pleomorphic cells of the hilus, being more evident in the soma, nucleus excluded, although it is also detectable in the neuropil, including both dendrites and axons (Figures 1C–F). Astroglial occurrence of CaSR was rather rare and when detected was of very low intensity as compared to neurons; appearing as puncta mainly restricted to the astrocytic soma (Figures 1K–P).

### Increase of CaSR expression in 3xTg-AD animals

**Figure 2:**
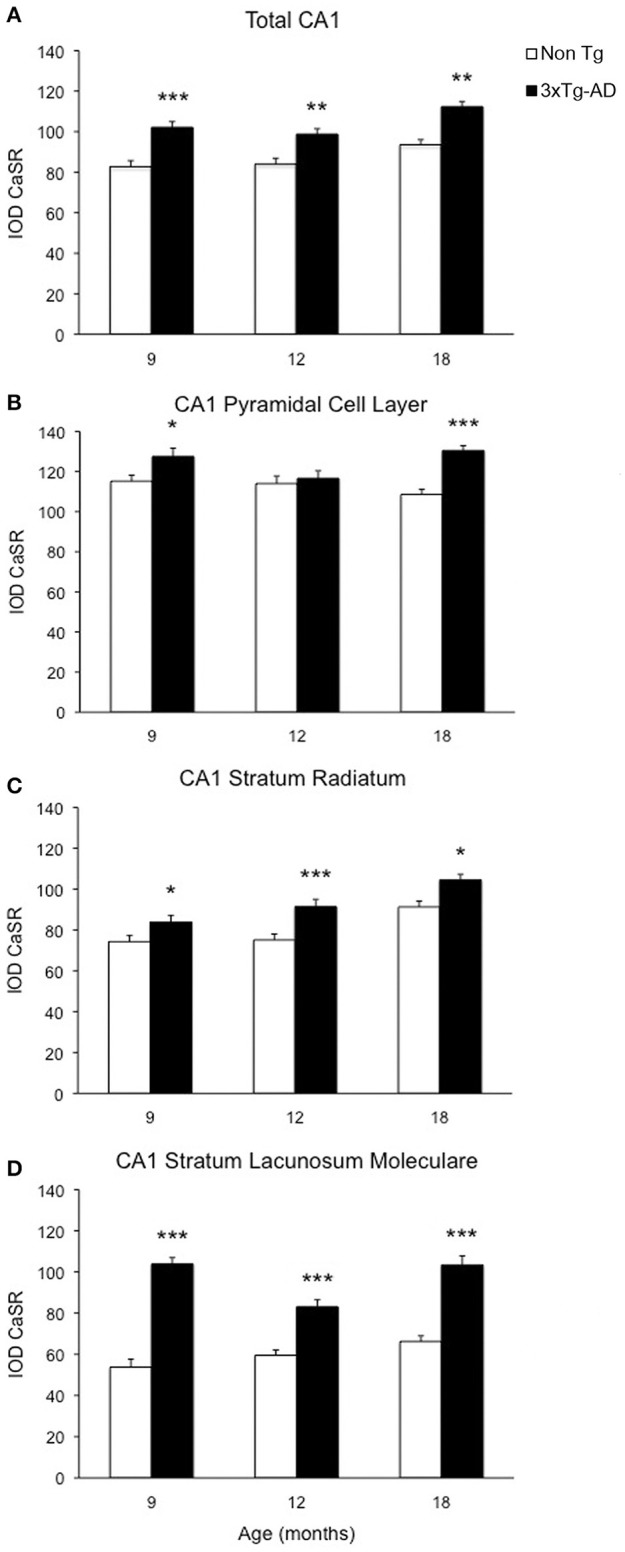
**Changes of CaSR expression in hippocampal CA1**. Bar graphs illustrating CaSR-IR IOD within CA1 subfield of hippocampus between non-Tg controls and 3xTg-AD mice at 9, 12 and 18 months of age (Ns for non-Tg = 5, 5, 4; ns for 3xTg-AD = 4, 5, 3, respectively; 3 slices have been analyzed per each animal). **(A)** Total CA1, **(B)** CA1 Pyramidal Cell Layer, **(C)** CA1 Stratum Radiatum, **(D)** CA1 Stratum Lacunosum-Moleculare. Bars represent means ± SEM (^*^p ≤ 0.05, ^**^p ≤ 0.01, ^***^p ≤ 0.001).

**Figure 3:**
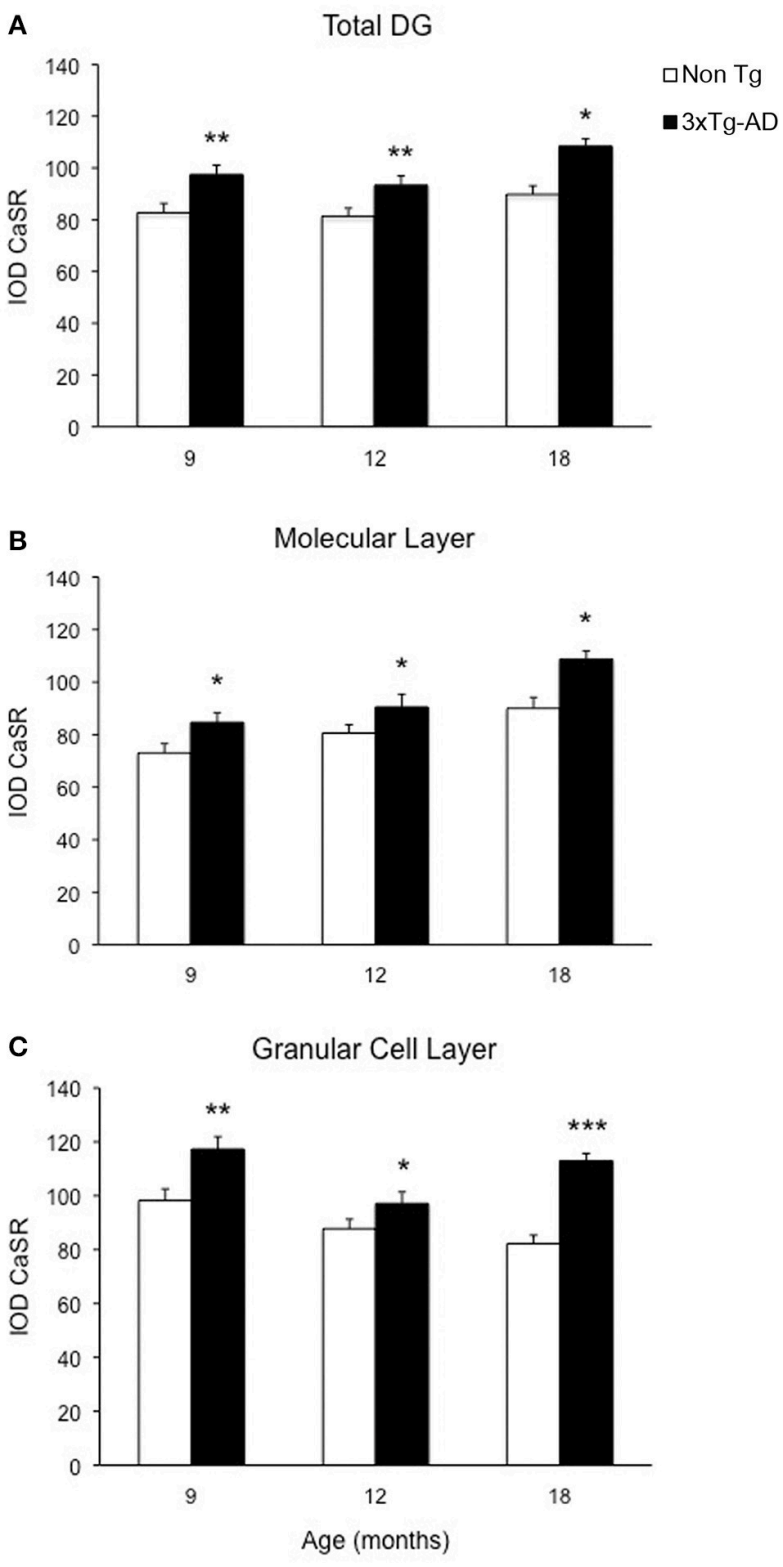
**Modification of CaSR expression in hippocampal DG**. Bar graphs illustrating CaSR-IR IOD within DG subfield between non-Tg controls and 3xTg-AD mice at 9, 12, and 18 months of age (Ns for non-Tg = 5, 5, 4; ns for 3xTg-AD = 4, 5, 3, respectively; 3 slices have been analyzed per each animal). **(A)** Total DG; **(B)** Molecular Layer; **(C)** Granular Cell Layer. Bars represent means ± SEM (^*^p ≤ 0.05, ^**^p ≤ 0.01, ^***^p ≤ 0.001).

Optical density analysis of CaSR staining in the hippocampus of the 3xTg-AD mouse showed a significant increase in its expression in CA1 and DG subfields [*F*_(7, 166)_ = 11.32, *p* < 0.0001 and *F*_(7, 166)_ = 7.994, *p* < 0.0001, respectively] at all ages (9, 12, and 18 months; Figures 1, 2). An increase in CaSR optical density of 23.44, 17.62, and 20.09% at 9, 12, and 18 months of age respectively when compared to control non-Tg animals was detected in CA1 area (Figures 1C,D, 2A; Table 1). Similar increase in CaSR optical density was observed in DG: 17.82, 14.74, and 20.74%, at 9, 12, and 18 months respectively in 3xTg-AD animals compared to non-Tg (Figures 1E,F, 3A; Table 1). No significant alterations in the optical density of CaSR were identified in the CA2 and in CA3 sub-fields.

**Table 1:** **Summary of CaSR IOD values in the hippocampal CA1 and DG subfields and their respective layers in both non-Tg and 3xTg-AD**.

**CaSR IOD**	**9 months NTG**	**9 months 3xTG-AD**	**12 months NTG**	**12 months 3xTG-AD**	**18 months NTG**	**18 months 3xTG-AD**
Total CA1	82.71 ± 2.98	102.1 ± 2.72^***^	83.92 ± 2.51	98.71 ± 2.56^***^	93.51 ± 3.39	112.30 ± 4.68^**^
PCL CA1	115.20 ± 3.78	127.50 ± 3.80^*^	114.00 ± 2.49	116.60 ± 2.39*ns*	108.60 ± 4.57	130.50 ± 3.40^**^
St Rad CA1	74.23 ± 2.91	83.83 ± 3.60^*^	75.08 ± 2.85	91.38 ± 2.75^***^	91.23 ± 3.31	104.5 ± 5.99^*^
St Lac CA1	53.78 ± 2.60	103.9 ± 3.33^***^	59.53 ± 2.71	83.09 ± 4.41^***^	66.25 ± 5.31	103.4 ± 6.25^***^
Total DG	82.68 ± 3.11	97.41 ± 3.56^**^	81.35 ± 3.33	93.34 ± 2.80^**^	89.78 ± 4.75	108.40 ± 4.96^*^
ML	73.06 ± 3.16	84.68 ± 4.88^*^	80.58 ± 4.18	90.51 ± 3.01^*^	90.00 ± 4.11	108.8 ± 6.91^*^
GCL	98.30 ± 3.55	117.30 ± 4.27^**^	87.76 ± 3.15	97.13 ± 2.78^*^	82.18 ± 6.16	112.90 ± 3.54^***^

Values are expressed as means ± SEM (

* *p ≤ 0.05*,

** *p ≤ 0.01*,

*** *p ≤ 0.001)*.

### CaSR expression increase in CA1 and DG is layer-specific

In CA1 we found that the increase in CaSR expression in 3xTg-AD animals mainly occurred in the pyramidal cell layer (PCL, 10.68% at 9 months, 2.28% at 12 months, 20.17% at 18 months; Figure 2B), in the stratum radiatum (SR, 12.93% at 9 months, 27.71% at 12 months, 14.55% at 18 months; Figure 2C) and in the stratum lacunosum moleculare (SLM, 93.19% at 9 months, 39.58% at 12 months, 56.08% at 18 months; Figures 1G,H, 2D; Table 1).

In the DG an increase in CaSR expression was most prominent in the molecular layer (ML) of 3xTg-AD animals respect to their controls (15.90% at 9 months, 12.32% at 12 months, 20.98% at 18 months; Figure 3B) and in the granular cell layer (GCL, 19.33% at 9 months, 10.68% at 12 months, 37.38% at 18 months; Figures 1I,J, 3C; Table 1). The increase in the molecular layer was similar in all subdivisions; the inner, middle and outer ML (data not shown).

Increased level of CaSR labeling in the projections layers described above doesn't appear to derive from neuronal somata, despite its presence in some interneurons cell bodies, but mainly from the neuropil comprising both dendrites and axons (Figures 1D,F).

### CaSR increase in hippocampus occurs in the areas affected by β-amyloid accumulation

**Figure 4:**
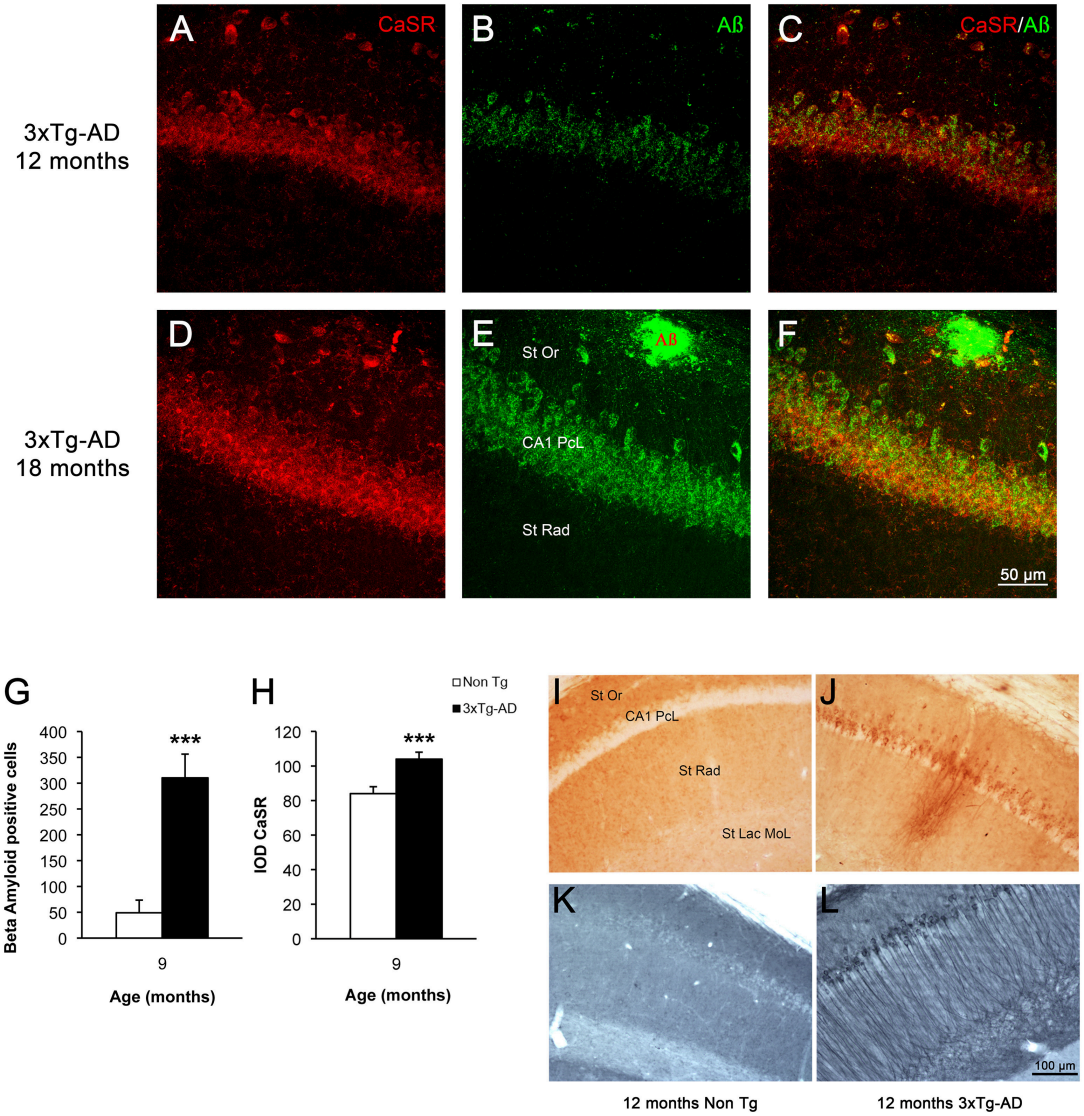
**CaSR and β-amyloid relationship in hippocampus during AD progression**. Confocal micrographs showing the overlapping expression of CaSR (red) with the accumulation of intraneuronal Aβ (green, anti-Aβ 488) and the formation of plaques in hippocampal pyramidal neurons of 3xTg-AD mice of 12 and 18 months of age respectively. **(A,D)** CaSR-IR; **(B,E)** Aβ-IR; **(C,F)** merge. St Or Stratum Oriens; CA1 PcL CA1 Pyramidal Cell Layer; St Rad Stratum Radiatum. Bar graphs illustrating the number of Aβ positive cells **(G)** and the IOD of CaSR **(H)** within CA1 subfield of hippocampus between non-Tg controls and 3xTg-AD mice at 9 months of age (^***^p ≤ 0.001). Brightfield micrographs of CA1 areas of hippocampus of non-Tg and 3xTg-AD mice immunolabeled with anti-Aβ **(I,J)** and anti-Tau antibody **(K,L)** at 12 months of age. St Or, Stratum Oriens; CA1 PcL, CA1 Pyramidal Cell Layer; St Rad, Stratum Radiatum; St Lac Mol, Stratum Lacunosum Moleculare.

In 3xTg-AD mice intracellular β-amyloid in hippocampal neurons appears between 4 and 6 months of age and reaches its maximum at 9 months of age (Figures 4G,H); extracellular β-amyloid depositions start to mount from 9 to 12 months, being maximal at 18 months. At 18 months the β-amyloid plaques are big and expanded through the hippocampus mainly concentrating in the CA1 subfield (Figures 4E,F,I,J); with later appearance in the DG (Noristani et al., 2012; Rodríguez et al., 2013). In a similar way hyper-phosphorylated Tau protein begins to be detectable in 3xTg-AD mice at 12 months causing the formation of tangles by 18 months, and it is also concentrated in CA1 region affecting all the layers (Figures 4K,L). The increase of CaSR intensity in the CA1 region follows the same spatio-temporal pattern as that of β-amyloid deposition (Figures 4A–F), while in the DG it appears even before the accumulation of Aβ becomes evident.

## Discussion

*In vitro* exposure of human cortical astrocytes and neurons to a truncated form of β-amyloid, the Aβ_25–35_ peptide, which binds and activates the CaSR (Dal Prà et al., 2014), stimulated the intracellular production and secretion of β-amyloid by both cell types with the subsequent death of a fraction of neurons (Armato et al., 2013). While neurons were vulnerable to β-amyloid toxicity, cultured astrocytes survived and they showed a transient increase of CaSR expression. The cytotoxic effects on neurons were fully inhibited following addition of CaSR antagonist NPS 2143 to the incubation media. Indeed, the NPS prevented the death of neurons and it deeply and steadily downregulated the receptor in the Aβ-treated astrocytes, and completely blocked the Aβ self-induction mechanism (Armato et al., 2013).

This *in vitro* study conceptually complements results we obtained in the 3xTg-AD mice model described in the present paper; these results further corroborate links existing between increases in CaSR expression and β-amyloid accumulation. In the 3xTg-AD mice, intraneuronal β-amyloid starts to accumulate in the CA1 pyramidal neurons at 4–6 months of age reaching maximum at 9 months of age, while extracellular β-amyloid deposits emerge from 12 months of age. Appearance of senile plaques is associated with specific astrogliosis around the plaques despite the generalized hippocampal astrocytic atrophy and with an increase in the density of activated microglia and recruitment of new ramified microglial cells (Rodríguez et al., 2009, 2010, 2013; Olabarria et al., 2010). Accumulation of Tau that occurs later follows similar pattern with maximum presence from 18 months. The 3xTg-AD mouse model not only develops the typical histopathological hallmarks of Alzheimer's disease, but also manifests synaptic dysfunction with impaired long-term-potentiation (LTP) (Oddo et al., 2003b; Rodríguez et al., 2008).

Analysis of an acute hypoxia/ischemia in rats revealed that Aβ overproduction due to hypoxic injury is mediated by CaSR hyperexpression (Bai et al., 2015). Experiments on rat hippocampal neurons demonstrated that hypoxia induces an up-regulation of CaSR which in turn promotes the elevation of cytosolic [Ca^2+^] thereby producing an increase of BACE1 expression that results in the rise of Aβ_40_ and Aβ_42_ generation. In addition, specific inhibition of CaSR with Calhex 231, an allosteric antagonist of CaSR, induced a decrease of BACE1 and Aβ both *in vitro* and *in vivo* in hypoxic conditions (Bai et al., 2015). The prominent effect of the increased expression and activity of CaSR in hippocampus is confirmed also by analysing a mouse model of acute ischemic injury where a transient global cerebral ischemia (TGI) was induced. In this model CaSR was overexpressed in parallel with GABA_*B*_ receptor 1 downregulation and was followed by neuronal death. The administration of the calcilytic compound NPS89636 through intracerebroventricular injections after the TGI specifically blocked the activity of CaSR, restored GABA_B_R1 expression and protected hippocampal neurons from cell death. Furthermore, the treatment of TGI mice with calcilytics significantly improved learning and memory (Kim et al., 2014).

Altogether these findings reveal the importance and the impact which altered expression of CaSR has on maintaining the normal brain function, supporting the idea that its changes could contribute to the development and progression of pathologies such as Alzheimer's disease and stroke. Our present data in an AD mouse model show that expression of CaSR is increasing while neuropathology progresses. Previous works demonstrated that the administration of Aβ_42_ oligomers to neuronal and astrocytic human cell cultures increases the endogenous Aβ_42_ production and secretion resulting in a progressive death of neurons. This mechanism of Aβ self-induction, which contribute to the advance of AD, was demonstrated to be completely suppressed by adding a specific calcilytic like NPS 2143 to the cell culture (Armato et al., 2013; Dal Prà et al., 2015; Chiarini et al., 2016). It needs to be emphasized that the CaSR could be a promising target of investigation for not only further understanding AD pathology onset and spread but also for evaluating new therapeutic solutions.

## Author contributions

EG: Experimental work, data analysis, result preparation and writing of the manuscript. JR: Conception of the study, experimental design, data analysis, result preparation interpretation, discussion and writing of the manuscript. AV: Results interpretation and discussion and writing of the manuscript. AC, UA, and ID: Results interpretation and discussion.

### Conflict of interest statement

The authors declare that the research was conducted in the absence of any commercial or financial relationships that could be construed as a potential conflict of interest.

## Supplementary material

Supplementary Figure 1 **Brightfield micrographs showing the anatomy of the dorsal hippocampus in a section stained with toluidine blue (A)** and the delimited areas that were chosen in each single analyzed section as region of interest for the OD analysis in a representative section of a 3xTg-AD animal labeled for CaSR **(B)** as detailed in the Materials and Methods. **(C)** Micrograph showing the lack of staining in the negative control, after omission CaSR antibody.

## Abbreviations

AD: Alzheimer's disease
ANOVA: One-way Analysis of Variance
CaSR: Calcium-Sensing Receptor
DG: Dentate Gyrus
GCL: Granular Cell Layer
IOD: Inverted Optical Density
mGluR: metabotrobic Glutamate Receptors
ML: Molecular Layer
OD: Optical Density
PB: Phosphate Buffer
PCL: Pyramidal Cell Layer
S.Mol: Stratum Lacunosum-Moleculare
SO: Stratum Oriens
S.Rad: Stratum Radiatum
TS: Trizma-base Saline
VEGF: Vascular Endothelial Growth Factor
3xTg-AD: triple Transgenic mouse model of AD.
