# Supplementary figures and images for: Increased Calcium-Sensing Receptor Immunoreactivity in the Hippocampus of a Triple Transgenic Mouse Model of Alzheimer's Disease
Source: Front Neurosci. 2017 Feb 16;11:81. doi: 10.3389/fnins.2017.00081 (PMC5312420; doi:10.3389/fnins.2017.00081)

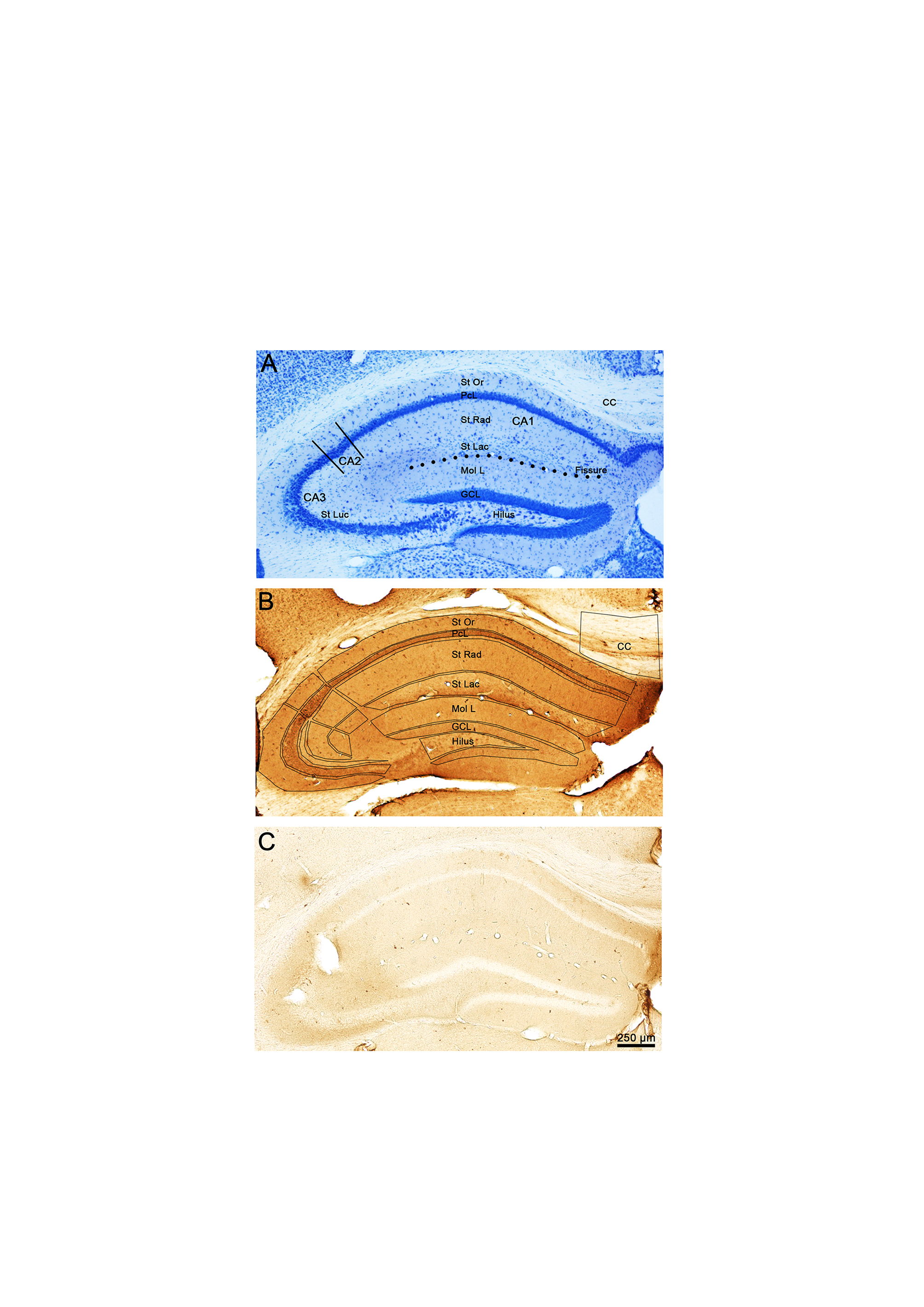

Supplement: Supplementary Figure 1 — Brightfield micrographs showing the anatomy of the dorsal hippocampus in a section stained with toluidine blue (A) and the delimited areas that were chosen in each single analyzed section as region of interest for the OD analysis in a representative section of a 3xTg-AD animal labeled for CaSR (B) as detailed in the Materials and Methods. (C) Micrograph showing the lack of staining in the negative control, after omission CaSR antibody. [file Image1.TIF]
